# 1,3,4-Thiadiazole Scaffold: As Anti-Epileptic Agents

**DOI:** 10.3389/fchem.2021.671212

**Published:** 2022-01-21

**Authors:** Tulika Anthwal, Sumitra Nain

**Affiliations:** Department of Pharmacy, Banasthali Vidyapith, Banasthali, India

**Keywords:** anti-convulsant, pharmacophoric pattern, 1,3,4 thiadiazole, structural activity relationship, anti-hypertensive

## Abstract

A wide range of biological activities is exhibited by 1,3,4-thiadiazole moiety such as antidiabetic, anticancer, anti-inflammatory, anticonvulsant, antiviral, antihypertensive, and antimicrobial. To date, drugs such as butazolamide, and acetazolamide. Several modifications have been done in the 1,3,4-thiadiazole moiety which showed good potency as anticonvulsant agents which are highly effective and have less toxicity. After in-depth literature survey in this review, we have compiled various derivatives of 1,3,4-thiadiazole scaffold as anticonvulsant agents.

## Introduction

Epilepsy is a CNS disease in which the activity of neuronal cells becomes abnormal, causing seizures, loss, or disturbance of consciousness with or without convulsion (Tripathi, 2013). In epileptic patients, a temporary disturbance in the messaging systems between brain cells is caused by a sudden surge of electrical activity due to which they experience recurrent seizures (Klein, 2019). Various factors are involved in the occurrence of epilepsy such as genetic factors, head trauma, conditions of the brain, infectious diseases, and prenatal injury to the brain. Electroencephalogram (EEG), CT scan, MRI, etc., are used to diagnose epilepsy seizures (Holland, 2018).

**FIGURE 1:**
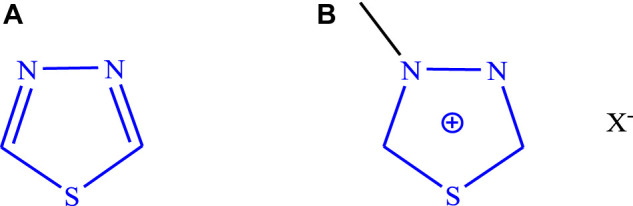
**(A)** Structure of 1,3,4-thiadiazole. **(B)** Mesoionic salts.

The molecular structure of a compound is responsible for various pharmacological activities, and mostly heterocyclic moieties have diverse activities. This scaffold **(** Figure 1A **)** derivatives possess a wide range of biological activities such as anticancer/antitumor (Janowska et al., 2020; Cevik et al., 2020; Gomha et al., 2017; Flefel et al., 2017; Ningegowda et al., 2017), anticonvulsant (Khatoon et al., 2018; Bhandari et al., 2008; Jatav et al., 2008; Foromadi et al., 2007; Almasirad et al., 2007; Dawood et al., 2006; Dogan et al., 2002; Varvaresou et al., 1998; Khazi et al., 1996; Stillings et al., 1986; Chapleo et al., 1986), antidiabetic (Vaishnav et al., 2017; Datar and Deokule, 2014; Thrilochana et al., 2014), anti-inflammatory (Cristina et al., 2018; Schenone et al., 2006), antidepressant (Can et al., 2012), antihypertensive (Samel and Pai, 2010), antiviral (Serban et al., 2020; Brai et al., 2019), antimicrobial (Gowda et al., 2020; Merugu et al., 2020; Mutchu et al., 2019; Sekhar et al., 2018; Joseph et al., 2015), antioxidant (Yakan, 2021; Gowda et al., 2020), anti-leishmanial (Tahghighi and Babalouei, 2017; Sadat-Ebrahimi et al., 2019), and neuroprotective (Skrzypeka et al., 2021). The biological activities of several synthesized derivatives of 1,3,4-thiadiazole are based on assumptions like “the = N-C-S- moiety’s presence and strong aromaticity of the ring, which is responsible for providing low toxicity and great *in vivo* stability. The derivatives of 1,3,4-thiadiazole have enormous capability to produce mesoionic salts **(** Figure 1B **)** and due to this behavior, they can interact strongly with biomolecules (proteins and DNA)” and can easily cross the blood–brain barrier (Haider et al., 2015; Serban et al., 2018).

**FIGURE 2:**
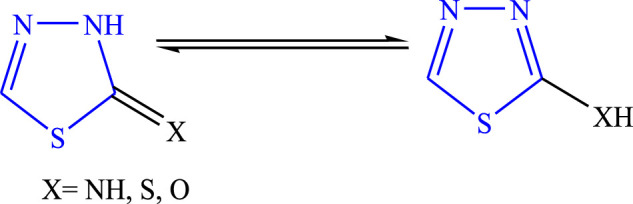
Tautomeric behavior of 1,3,4-thiadiazole.

**FIGURE 3:**
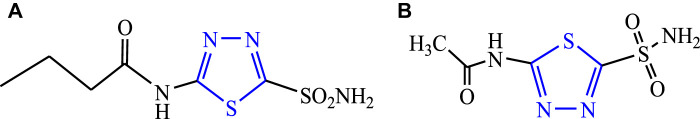
**(A)** Butezolamide. **(B)** Acetazolamide.

This scaffold was discovered in 1882 by Emil Fischer, although the true properties of the ring were described by Freund and Kuh. It is also known as 4-azathiazole or 3,4-dioxythiophene (Manimaran et al., 2017; Joseph et al., 2015). It is affected by the strong base and forms a ring cleavage when strong bases are added to it and become stable as acids are added to it. The tautomeric behavior (Figure 2) occurs due to 2-hydroxy-, mercapto-, and amino derivatives, and it is a pseudo-aromatic molecule in nature. The dipole moment of the moiety is 3.25 D; at the second and fifth positions, nucleophilic attack takes place. It is a stable colorless compound with a melting point of 42°C, it is potent for oxidation and reduction in alkali/acids and shows no ultraviolet absorption maximum up to 220 nm (Raj et al., 2015). Numerous drugs are available in the market of this moiety used for diverse biological activities such as butazolamide **(** Figure 3A **)**, acetazolamide **(** Figure 3B **)** (Loscher et al., 2011; Jain et al., 2013).

1,3,4-Thiadiazole moiety also has patent research on various activities such as potent agonist of the S1P1 receptor (Poveda et al., 2010), the antagonist of voltage-gated sodium channels (Stamos et al., 2009), proton pump inhibitor (Karimian et al., 1997), β-adrenergic blocking agents (Belanger, 1979).

### The Pharmacophoric Pattern of 1,3,4-Thiadiazole Responsible for Anticonvulsant Activity

A pharmacophore is a 3D structure to which several ligands can bind to the same protein in the same binding site by observing their common arrangements of ligands to identify a biologically active compound.

Nowadays, the most applicable way to design a new drug molecule that has a high affinity to bind with a specific receptor to give appropriate results is none other than a pharmacophore-based approach (Raj et al., 2015).

The thiadiazole ring exhibits various biological activities, but there are various important features due to which the thiadiazole ring acts as an anticonvulsant agent such as “a hydrogen bonding domain (HBD), Hydrophobic aryl ring (Ar), another distal Hydrophobic site, an electron-donor group (D)” (Pandey, 2002).

### Mechanism of Action of 1,3,4-Thiadiazole in Treatment of Epilepsy

There are several non-synaptic and synaptic mechanisms which are responsible for treatment of epilepsy. Literature survey reveals the importance of GABA receptors in the mechanism and treatment of epilepsy by releasing chloride ions and preventing the abnormal electrical impulse in the brain. The essential features of this moiety which is responsible for anticonvulsant activity include an electron-donor group, a hydrophobic aryl ring, a distal hydrophobic site, and a hydrogen bonding domain. 1,3,4-Thiadiazole prevents neurons from firing in the brain by releasing the chloride ions due to the GABAA pathway (Khatoon et al., 2018; Bhattacharya et al., 2019).

### 1,3,4-Thiadiazole Derivatives as Anti-Convulsant

**FIGURE 4:**
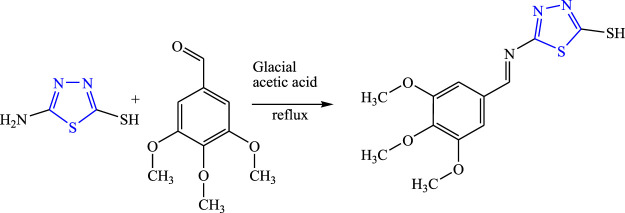
Systematic scheme for synthesizing the 5-amino-1,3,4-thiadiazole derivative.

A new Schiff-base derivative of 5-amino-1,3,4-thiadiazole was synthesized using glacial acetic acid and ethanol by the condensation method by Aliyu et al. **(** Figure 4 **)**. The synthesized compounds were characterized by analytical spectroscopy [FT-IR, NMR (^1^H ^13^C), MS, UV] and were evaluated for *in-vivo* anticonvulsant activity by maximal electroshock seizure (MES), phenobarbital-induced sleep test, and the rotarod method (neurotoxicity) using sodium valproate and phenytoin as the standard drug. Molecular docking studies were also performed using ChemSketch 1.21 software. The SAR activity revealed that the synthesized compound becomes more lipophilic and showed good antiepileptic activity. It concluded that the synthesized compound named 5-[(E)-(3,4,5-trimethoxybenzylidene)amino]-1,3,4-thiadiazole-2-thiol showed the H-bonding for the donor/acceptor group at a range of 4.18–6.88 Å. LD_50_ was found to be 3,807.87 mg/kg while it displayed 66.67% protection at 100 mg/kg for the MES method and 80% protection was observed at 100 mg/kg in the PTZ method. The compound was proven to be potent for both the method with no toxicity by two mechanisms GABA and the voltage-gated ion channel (Aliyu et al., 2021).

**FIGURE 5:**
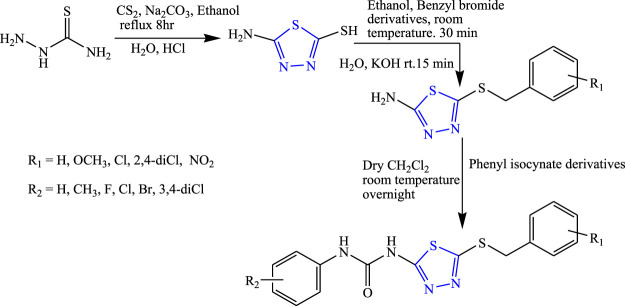
Systematic scheme for synthesizing 2,5-disubstituted 1,3,4-thiadiazoles derivatives.

Several derivatives of 1,3,4-thiadiazole were synthesized by substituting with phenyl isocyanate derivatives at the second and fifth positions, taking thiosemicarbazide and carbon disulfide as a precursor by Toolabi et al. **(** Figure 5 **)**. The synthesized compounds were characterized by analytical spectroscopy (IR, NMR, MS, HPLC) and were evaluated for *in-vivo* anticonvulsant activity by MES, phenobarbital-induced sleep test, and rotarod method using diazepam as the standard drug. Molecular docking was also performed using a BZD-binding pocket of the GABA receptor, and 11 compounds were found to be effective using AutoDock 4.2.1 software. The SAR activity of the compound concluded that an unsubstituted compound (i.e., compound containing hydrogen) showed good anticonvulsant activity as compared to the compound containing a halo group, while moderate activity was shown by a compound containing three methoxy groups. A decrease in activity was observed when the compound was substituted by two bromo and four methyl groups at phenyl urea. It is concluded that the Cl substitution on the benzyl thiol ring and Br substitution on the phenyl urea ring were responsible for good anticonvulsant activity. Two compounds named 1-{5-[(2,4-dichlorobenzyl)thio]-1,3,4-thiadiazol-2-yl}-3-(4 fluorophenyl) urea (ED_50_ = 2.70 and 0.65 μmol/kg) and 1-{5-[(3-methoxybenzyl)thio]-1,3,4-thiadiazol-2-yl}-3-phenylurea (ED_50_ = 2.72 and 1.14 μmol/kg) were found to be highly potent in sleep and MES test compared with the standard (Toolabi et al., 2020).

**FIGURE 6:**
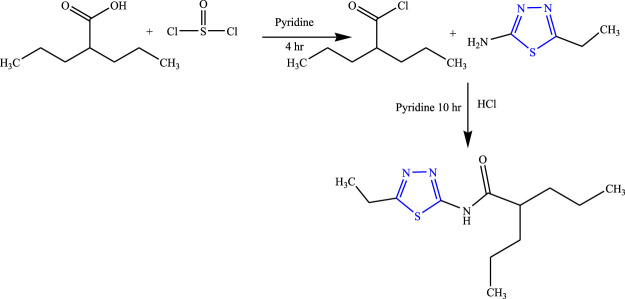
Systematic scheme for synthesizing the valproic acid analog.

A series of amide thiadiazole-linked valproic acid analog was synthesized by Malygim et al. **(** Figure 6 **)** and confirmed the structure using IR, NMR (^1^H, ^13^C), and MS while the purity was checked by using the TLC and HPTLC methods. The *in-vivo* anticonvulsant activity was checked using MES and pentylenetrazole-induced model in mice taking isoniazid and pentylenetetrazole as standard. It is concluded that the synthesized compound [N-(5-ethyl-1,3,4-thiadiazol-2-yl)-2-propyl pentane amide] when injected intraperitoneally was found to be 1.8 times more effective (LD_50_) than valproic acid, ED_50_ was 126.8 mg/kg, and the therapeutic index was found to be 7.3. The synthesized compound was found to be most effective for isoniazid-induced seizures (Malygin et al., 2020).

**FIGURE 7:**
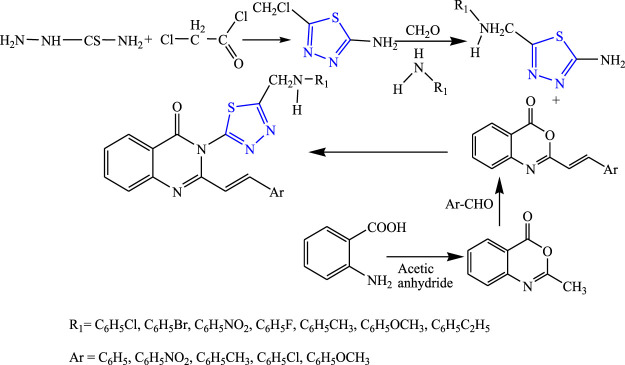
Systematic scheme for synthesizing derivatives of (*E*)-3-(5-(substituted amino methyl)-1,3,4-thiadiazol-2-yl)-2-styrylquinazolin-4(3H)-one.

A two-step reaction was carried out to synthesize several quinazolinone derivatives of 1,3,4-thiadiazole by reacting with 2-substituted benzoxazin-4-one by Bhattachara et al. **(** Figure 7 **)**. The spectral analysis of the synthesized compound was done, and *in-vivo* anticonvulsant activity was checked by scPTZ and MES models, taking phenytoin and carbamazepine as standard out of which four compounds were found most active. The compound named (E)-3-(5-{[(4-chlorophenyl)amino]methyl}-1,3,4thiadiazol-2-yl)-2-styryl quinazoline-4(3H)-one showed the highest potency at 30 mg/kg within 30 min. The SAR studies concluded that compounds containing nitro and chloro groups displayed potent anticonvulsant activity (Bhattacharya et al., 2019).

**FIGURE 8:**
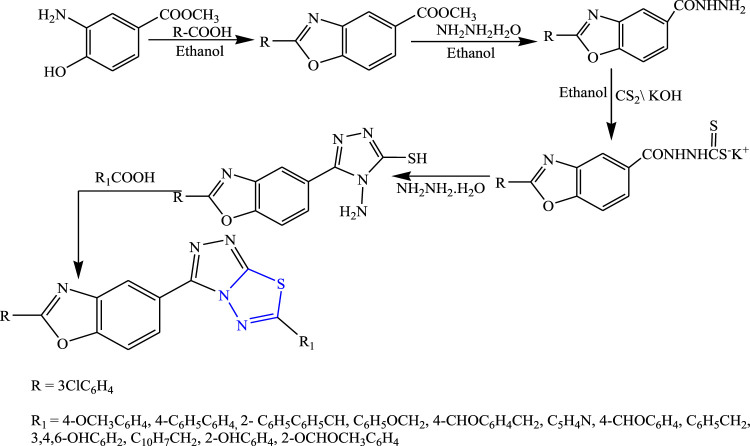
Systematic scheme for synthesizing 1,2,4-triazole-1,3,4-thiadiazole derivatives.

Several 1,3,4-thiadiazole derivatives were synthesized by condensing 3-amino-4-hydroxybenzoate along with ethanol to obtain the main compound **(** Figure 8 **)** by Sarafroz et al. The characterization of the synthesized compound was done by NMR and IR and its *in-vivo* anticonvulsant activity was checked by scPTZ and MES models, and for determining the neurotoxicity the rotarod method was used, although all compounds (1,2,4-triazole-1,3,4-thiadiazoles substituted amino derivatives) showed good anticonvulsant activity; three compounds were found to be highly potent at 30 mg/kg within 30 min. SAR activity revealed that the aldehyde and hydroxy-substituted groups showed potent activity (Sarafroz et al., 2019).

**FIGURE 9:**
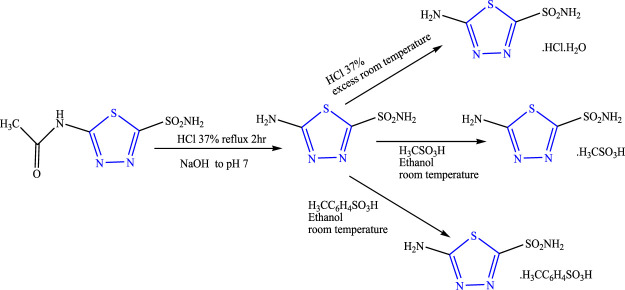
Systematic scheme for synthesizing 5-amino-2-sulfonamide thiadiazole salts.

A two-step reaction was performed to synthesize three different salts of thiadiazole using acetazolamide as a precursor in the presence of HCl and NaOH using ethanol as a solvent **(** Figure 9 **)** by Diaz et al. The characterization of the synthesized compound was done by spectroscopic techniques, and its *in-vivo* anticonvulsant activity was checked by intraperitoneally induced seizure using nikethamide and picrotoxins as standard. Neurotoxicity was determined by the rotarod method taking phenobarbital as the standard. *In-silico* studies were also performed in Hats. tosylate protein. It concluded that 5-amino-2-sulfonamide thiadiazole showed 72%–79% protection at 90 mg/kg against both the standard used when compared with its synthesized salts with no neurotoxicity; good binding affinity toward the selected protein by inhibiting carbonic anhydrase was also observed and was found effective against mild convulsions (Diaz et al., 2016).

**FIGURE 10:**
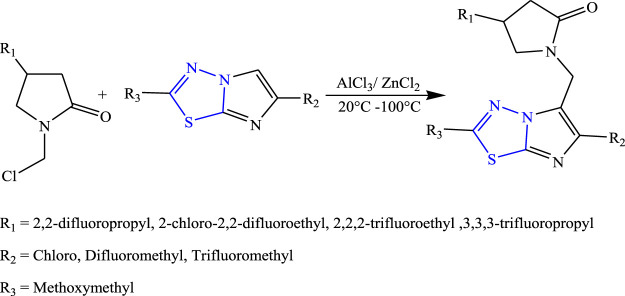
Synthetic pathway for designing 2-oxo-1-pyrrolidinyl imidazothiadiazole derivatives.

Several new 2-oxo-1-pyrrolidinyl imidazothiadiazole derivatives were synthesized by Quesnel et al. using Lewis acid as a reagent (Figure 10). The characterization of synthesized compounds was done by spectral analysis, and the compounds were screened for their *in-vivo* anticonvulsant profile using sound-susceptible mice (audiogenic seizures), Hz seizure model, and PTZ models. Although all the synthesized compounds showed good anticonvulsant activity, a compound named 4-(2,2-difluoropropyl)-1-{[2-(methoxymethyl)6-(trifluoromethyl) imidazo (2,1b) (1,3,4) thiadiazol-5-yl] methyl} pyrrolidine-2 one showed 95% anticonvulsant activity at the lowest dose by binding with SV2 protein. The SAR activity concluded that the addition of the F group increases the anticonvulsant activity of the synthesized compound (Quesnel et al., 2015).

**FIGURE 11:**
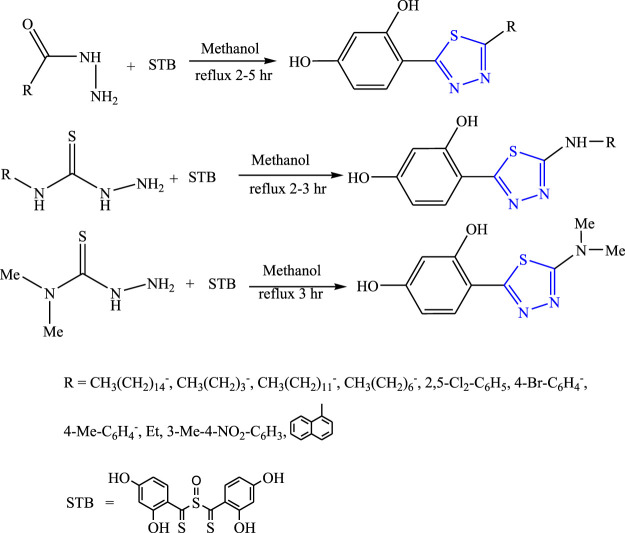
Synthetic pathway for designing thiosemicarbnyl/hydrazide derivatives of 1,3,4-thiadiazole.

Luszcksi et al. synthesized 13 new derivatives of 1,3,4-thiadiazol by refluxing thiosemicarbnyl/hydrazides and STB {sulfinylbis [(2,4-dihydroxyphenyl) methanethione]} in methanol (Figure 11). The characterization of synthesized compounds was done by the spectral analysis, and the compounds were screened for their *in-vivo* anticonvulsant profile by MES models using valproic acid as the standard drug. The SAR activity revealed that the substitution of long aliphatic chains with the ring either decreased the activity or showed no activity. Further, it concluded that only two compounds were found to be potent out of which 5-butyl-2-(2,4-dihydroxyphenyl)-1,3,4-thiadiazole (ED_50_ = 247- >500 mg/kg within 15 min) was found to be a highly potent compound (Luszczki et al., 2014).

**FIGURE 12:**
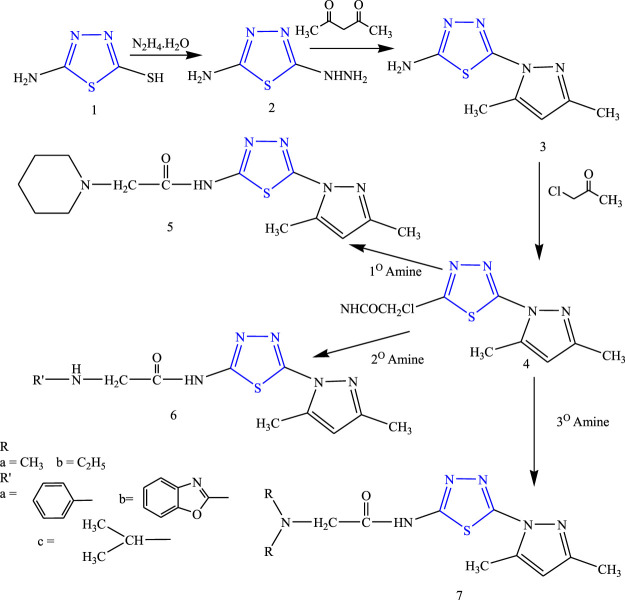
Synthetic pathway for designing amide 1,3,4-thiadiazole derivatives.

Some new thiadiazole derivatives **(** Figure 12 **)** were synthesized by refluxing compound 1) and hydrazine hydrate (ethanolic solution) to form compound 2), then to the reaction mixture carbon disulfide and acetylacetone were added to form compound 3), then the equimolar quality of chloroacetyl chloride was added to form the acetamide complex 4) which acts as the main intermediate, to which the equimolar amount of ethanolic solution of primary, secondary, and tertiary amine was added; their anticonvulsant activity was reported by Rahman et al. After spectral analysis, the synthesized compounds were screened for their anticonvulsant profile on albino mice. Although all the synthesized compounds showed good anticonvulsant activity, a compound named 2-(diethylamino)-N-(3,5-dimethyl-1H-pyrazol1-yl)-1,3,4-thiadiazole-2-yl acetamide exhibited 50% or more prominent activity against induced convulsion at a lower dose of 30 mg/kg at 30 min. It concluded that the lipophilic nature of the ring is responsible for the activity (Rahman et al., 2014).

**FIGURE 13:**
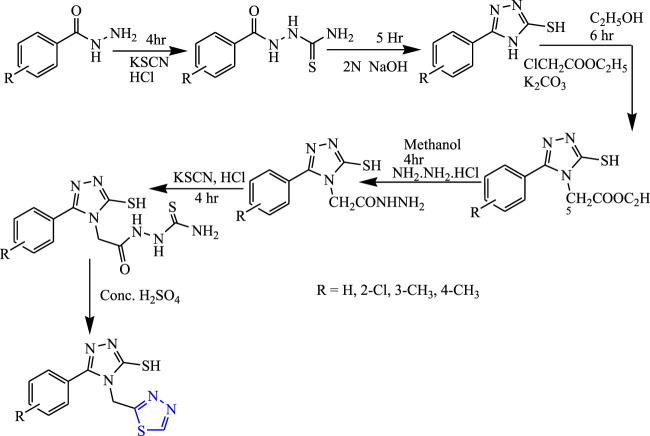
Systematic scheme for synthesizing compounds by substituting 1,3,4-thiadiazole with Phenyl hydrazides and Benzoic acid.

Several 1,3,4-thiadiazole derivatives were synthesized from substituted phenyl hydrazides and substituted benzoic acid using alcohol and water as a solvent (Figure 13) by Kumudha et al. The characterization of synthesized compounds was done by spectral analysis, and the compounds were screened for their *in-vivo* anticonvulsant profile using PTZ and MES models. All the synthesized compounds were found to be potent, but compounds named 4-[(1,3,4-thiadiazol-2-yl)methyl]-5-p-tolyl-4H-1,2,4-triazole-3-thiol showed good activity during the PTZ and MES tests (83% and 75% inhibition) at 20 mg/kg with less toxicity. It concluded that the electron-substituted group showed better anticonvulsant activity (Kumudha et al., 2014).

**FIGURE 14:**
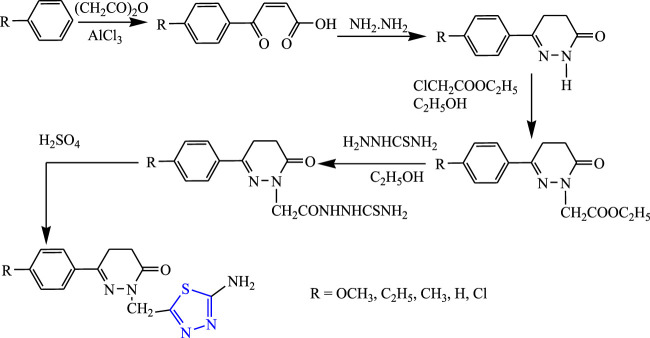
Systematic scheme for synthesizing 5-amino-1,3,4-thiadiazole derivatives.

Several 1,3,4-thiadiazole derivatives were synthesized by Sharma et al. Friedel craft acylation was done to obtain the intermediate b-aroyl propionic acid, aluminum chloride, and C_4_H_4_O_3_ which were further treated with hydrazine hydrate from pyridazine and then treated with ethyl chloroacetate to form acetic acid ethyl ester, then thiosemicarbazide was added and on stirring to form hydrazine carbothioamide, and then it was treated with concentrated sulfuric acid to synthesize the final compound **(** Figure 14 **)**. Their characterization of synthesized compounds was done by NMR, IR, and mass spectral analyses, and the *in-vivo* anticonvulsant activity was checked by MES and scPTZ models taking diazepam as standard out of which two compounds were found most active. The compound named {2-[(5-amino-1,3,4-thiadiazol-2-yl)methyl]-6-(4chlorophenyl)-4,5-dihydropyridine-3(2H)-one} showed 85.44% inhibition in both scPTZ (100 mg/kg) and MES (50 mg/kg) tests; it further concluded that the Cl substituent compound was found to be effective (Sharma et al., 2014).

**FIGURE 15:**
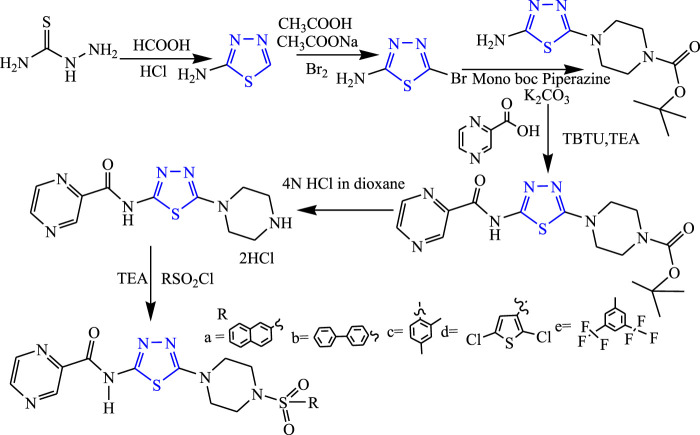
Systematic scheme for synthesizing substituted 1,3,4-thiadiazole pyrazines derivatives.

Several derivatives of substituted 1,3,4-thiadiazole pyrazine were synthesized by Kikkeri et al. **(** Figure 15 **)**. They evaluated *in-vivo* anticonvulsant activity using the MES method, and neurotoxicity was checked using the rotarod method taking phenytoin as standard. Two compounds named N-(5-{4-[(2,5-dichlorothiophen-3-yl)sulfonyl]piperazine1-yl}-1,3,4-thiadiazole-2-yl)pyrazine-2-carboxamide (74.52% inhibition) and N-(5-(4-{[3,5 bis (trifluoromethyl) phenyl]sulfonyl} piperazine-1-yl)-1,3,4-thiazol-2-yl) pyrazine-2-carboxamide (74.88% inhibition) were found to be active. The SAR study of these compounds indicated that more anticonvulsant activity is observed when the phenyl ring is introduced as compared to the methyl group (Kikkeri et al., 2013).

**FIGURE 16:**
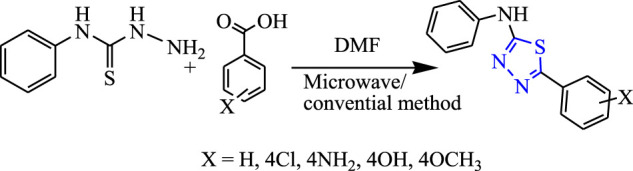
Synthetic pathway for designing phenyl-1,3,4-thiadiazol-2-amine derivatives.

Sahoo et al. synthesized 1,3,4-thiadiazole derivatives by both conventional and microwave-irradiated methods **(** Figure 16 **)**. The microwave method was more effective and gave a high yield. The characterization of compounds that were newly synthesized was done by ^1^H NMR, IR, and LC-mass, and an *in-vivo* study was done by the MES method while a neurotoxicity study was done by the rotarod test using phenytoin as a standard compound. Compounds substituted by the OCH_3_ group [5-(3-methoxyphenyl)-N-phenyl-1,3,4-thiadiazol-2-amine] were found to be highly effective; it showed 19.64% protection at a low dose (30 mg/kg) and 64.28% protection at a high dose (300 mg/kg) with no toxicity. Molecular docking was performed targeting voltage-gated channels using Glide XP software (Sahoo et al., 2013).

**FIGURE 17:**
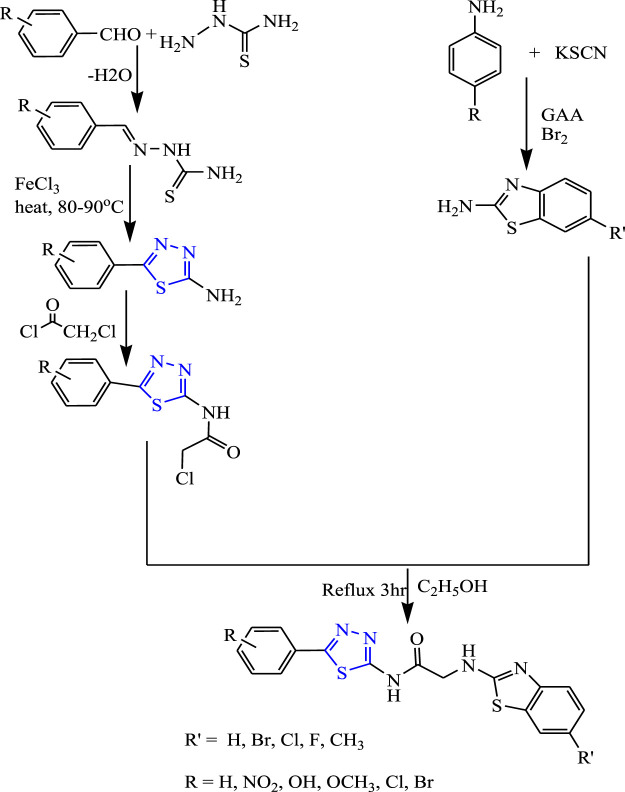
Systematic scheme for synthesizing 1,3,4-thiadiazol-acid-amide derivatives.

Several Schiff-base 1,3,4-thiadiazol-acid-amide derivatives **(** Figure 17 **)** were synthesized by Siddiqui et al. An important role was played by the benzothiazole hydrophobic domain through amino acetamide linkage for anticonvulsant activity. The synthesized compounds were characterized by spectral analysis; an *in-vivo* activity was studied by the MES method, and a neurotoxicity study was done by the rotarod method using phenytoin as a standard compound. Two compounds 2-[(6-fluoro-1,3-benzothiazole-2-yl)amino]-N-[5-(4-nitrophenyl)-1,3,4-thiadiazol-2-yl] acetamide and N-[5-(4-methoxyphenyl)-1,3,4-thiadiazol-2-yl]-2-[(6-methyl-1,3-benzothiazole-2-yl)amino] acetamide were found to be highly effective as they showed 100% protection at a low dose (30 mg/kg) with no toxicity at a low dose (30 mg/kg) at 30 min (Siddiqui et al., 2013).

**FIGURE 18:**
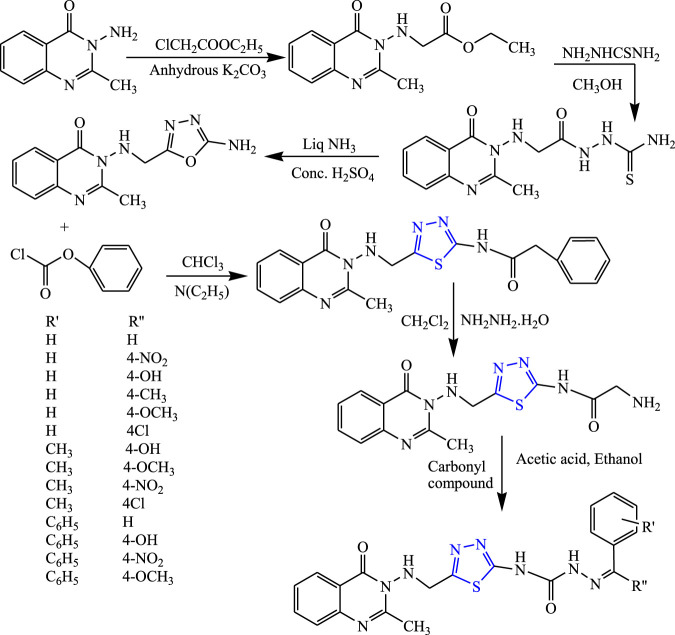
Systematic scheme for synthesizing 1,3,4-thiadiazole quinazoline analogs.

Rajak et al. synthesized semi-carbazones from 3-amino-2-methyl quinazoline-4(3H)-ones by refluxing them for 6–8 h **(** Figure 18 **)**. The characterization of newly designed compounds was done by IR and NMR, and *in-vivo* activity was studied using PTZ and MES models. Although all the compounds were found to be potent, N-(5-({[2-methyl-4-oxoquinazolin-3(4H)-yl] amino} methyl)-1,3,4-thiadiazol-2-yl)-N^4^-[1-(4nitrophenyl) (phenyl) methanone]-semicarbazone was found to be highly potent for both models (100 mg/kg for MES and 300 mg/kg for PTZ). The SAR study of the synthesized compounds concluded that the p-substituted group present in aryl moiety changes variation on the activity (anti-convulsion) of the test compounds and a compound containing the methyl group showed potent activity while the compound substituted with chloro and nitro groups displayed the highest potency (Rajak et al., 2012).

**FIGURE 19:**
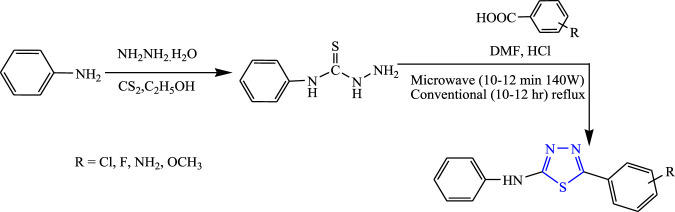
Synthetic pathway for designing phenyl-1,3,4-thiadiazol-2-amine derivatives.

Several new derivatives of 1,3,4-thiadiazole were synthesized by Sahoo et al. by both microwave-irradiated and conventional methods. Cyclization of various aromatic acids with N-phenyl thiosemicarbazide was done to obtain the main compounds **(** Figure 19 **)**. The microwave method was more effective and gave a high yield. The synthesized compounds were characterized by spectral analysis, and an *in-vivo* study was done using the MES model in rats. 5-(4-Methoxyphenyl)-N-phenyl-1,3,4-thiadiazol-2-amine showed maximum protection (64.28%) at 300 mg/kg (Sahoo et al*.*, 2012).

**FIGURE 20:**
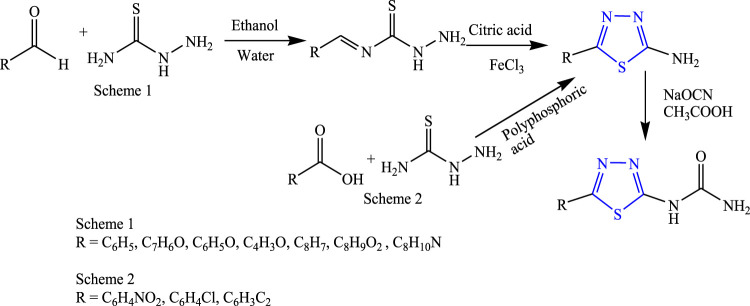
Systematic scheme for synthesizing aryl-substituted 1,3,4-thiadiazole analog.

Several new 1,3,4-thiadiazole derivatives were designed by Gowramma et al. first by doing oxidative cyclization of thiosemicarbazone and citric acid to get the intermediate, and then the final compound was obtained by adding sodium cyanate in glacial acetic acid using ethanol and water as a solvent and refluxing at 80°C–90°C for 45 min **(** Figure 20 **)**. The characterization of synthesized compounds was done by NMR and IR, and *in-vivo* anticonvulsant activity was checked by subcutaneous pentylenetetrazole (scPTZ) taking phenytoin as standard. A compound containing nitro and chloro groups showed prominent anticonvulsant activity at 100 mg/kg (Gowramma et al., 2012).

**FIGURE 21:**
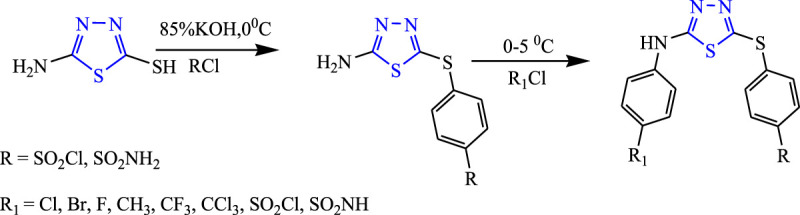
Systematic scheme for synthesizing amino 1,3,4-thiadiazole analogs.

Several 1,3,4-thiadiazole analogs were synthesized from an amino 1,3,4-thiadiazole derivative by Sharma et al. **(** Figure 21 **)** by stirring 85% KOH solution and 4-chloro benzene sulfonamide at 0°C and 5°C, and then the intermediate was added to obtain the final compound using ether. The characterization of the design compounds was done by spectral analysis, and their *in-vivo* study was done using PTZ and MES; five compounds showed good anticonvulsant activity, but the compound named 4-[5-(4-trifluoromethyl-phenylamino)-(1,3,4)thiadiazol-2-ylsulfanyl]-benzene sulfonyl chloride showed 66.6% inhibition in the MES model and zero death in the PTZ model (Sharma et al., 2011).

**FIGURE 22:**
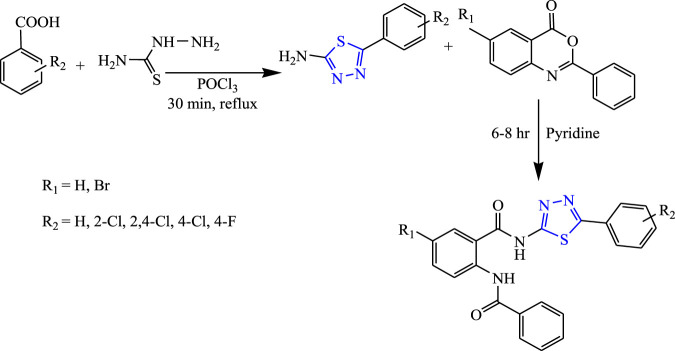
Systematic scheme for synthesizing carboxamide 1,3,4-thiadiazole derivatives.

Several analogs of 1,3,4-thiadiazoles containing carboxamide nucleus were synthesized by Masi et al. by condensing 2,5-disubstituted-1,3,4-thiadiazole with benzoxazine **(** Figure 22 **)**. The characterization of the design compounds was done by spectral analysis, and *in-vivo* study was done using the PTZ model taking carbamazepine as standard. Although all the compounds showed good results, the bromo-substituted compounds were found to be potent especially 2-benzamide-5-bromo-N-[5-(2-chlorophenyl)-1,3,4-thiadiazol-2-yl] benzamide showed 100% protection at 60 mg/kg for mortality (1–24 h); therefore, it is concluded that substitution with Br increases the activity of the compound (Masi et al., 2011).

**FIGURE 23:**
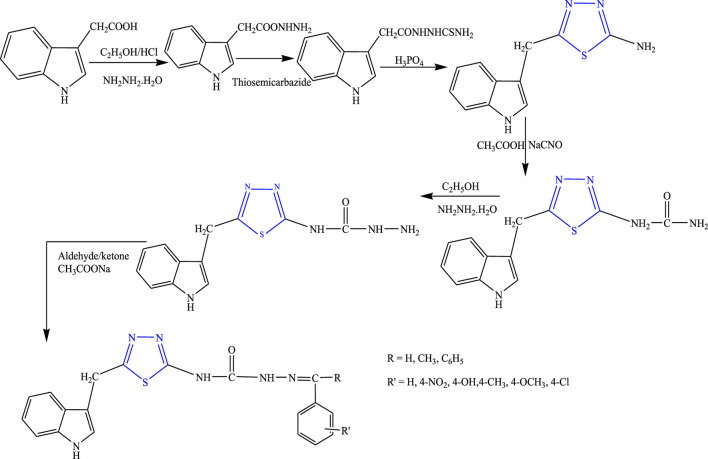
Synthetic pathway for designing 1,3,4-thiadiazoles derivatives.

Rajak et al. designed di-substituted derivatives of 1,3,4-thiadiazoles (Figure 23). The characterization of the synthesized compounds was done by spectral analysis, *in-vivo* study was done using the PTZ and MES methods, and neurotoxicity was checked by the rotarod test. Among the synthesized compounds, one compound named N^1^-[5-(1H-indol-3-ylmethyl)-1,3,4-thiadiazol-2yl]-N^4^-[1-(4-hydroxyphenyl) (phenyl) methanone] semicarbazone was found to be the most potent compound as it was effective for both PTZ (300 mg/kg) and MES (100 mg/kg) methods without any neurotoxicity. The SAR study reveals that if the nitro group and the hydroxy group are introduced on the phenyl ring, it showed high-potency *in-vivo* tests (Rajak. et al., 2010).

**FIGURE 24:**
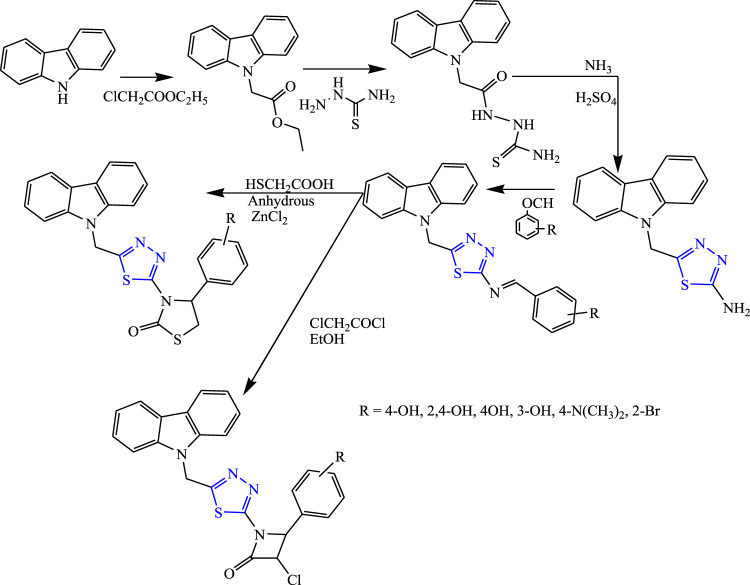
Systematic scheme for synthesizing thiadiazolylazetidinonyl carbazole derivatives.

Several substituted thiadiazolylazetidinonyl carbazole derivatives were synthesized by Kaur et al. **(** Figure 24 **)**. The characterization of the synthesized compounds was done by spectral analysis, and *in-vivo* study was done using the PTZ and MES methods. The neurotoxicity of the compounds was checked using the rotarod test. Only one compound (3-{5-[(9H-carbazol-9-yl) methyl]-1,3,4-thiadiazol-2-yl}-4-(3-bromophenyl) thiazolidine-2-one) showed potent results at 40 mg/kg with 90% protection for the MES test (LD_50_ > 1,600) (Kaur et al., 2010).

**FIGURE 25:**
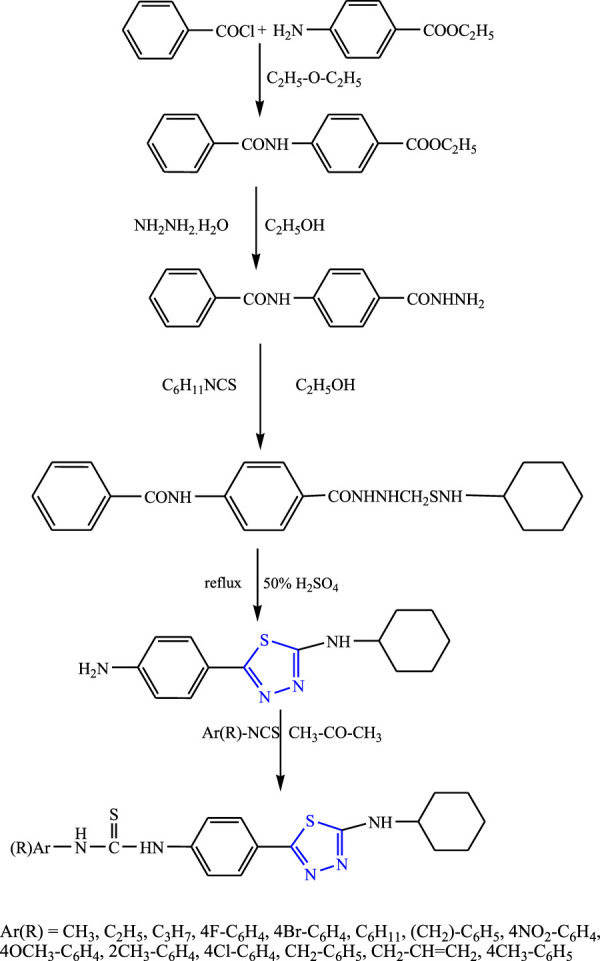
Systematic scheme for synthesizing 5-cylohexylamino-1,3,4-thiadiazole derivatives.

Several new derivatives of 5-cylohexylamino-1,3,4-thiadiazole were synthesized taking benzoyl chloride and ethyl 4-aminobenzoate as precursors by Karakus et al. **(** Figure 25 **)**. The characterization of the compounds was done by IR and ^1^H-NMR, and an *in-vivo* study was done using PTZ and MES methods. The SAR studies indicate that groups (chloro, allyl, methyl) attached to phenyl were responsible for increasing the activity of the compound. It concluded that although all the compounds showed good activity against petit mal seizures, the compound named N-{4-[(5-cyclohexylamino)-1,3,4-thiadiazole-2-yl]phenyl}N9-(2-methylphenyl) thiourea showed the highest protection (68.42%) at 25 mg/kg (Karakus et al., 2009).

**FIGURE 26:**
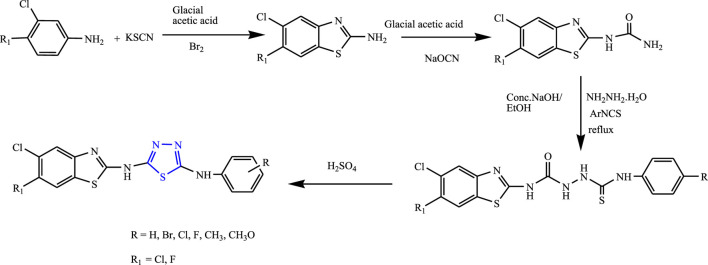
Synthetic pathway for designing 1,3,4 thiadiazole derivatives.

Several derivatives of 1,3,4-thiadiazoles were synthesized from substituted acetophenones by Siddiqui et al. **(** Figure 26 **)**. The characterization of the compounds was done by spectral analysis, and an *in-vivo* study was done using PTZ and MES using phenytoin sodium as standard. Two compounds were found to be potent for the MES test, out of which N-(4-chlorophenyl)-N^5^-[5,6-dichlorobenzo(d)thiazol-2-yl]-1,3,4-thiadiazole-2,5-diamine was found to be highly effective (100% protection at 30 mg/kg with no toxicity). It is concluded that the compound containing the halo group showed good anticonvulsant activity when compared to another group attached (Siddiqui et al., 2009).

**FIGURE 27:**
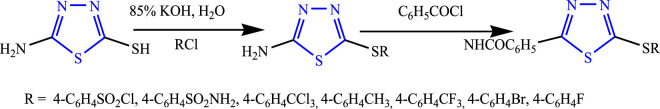
Systematic scheme for synthesizing amine-1,3,4-thiadiazole analog.

Seven new thiadiazole derivatives were synthesized by Pattanayak et al. by dissolving 5-sulfanyl-1,3,4-thiadiazole-2-arylamine in 85% KOH solution and then stirring it for 5–10 min at room temperature, then the temperature was brought down to 0°C. An equimolar amount of aromatic halide (R) was added with vigorous stirring to form an intermediate, then a mole intermediate was dissolved in distilled water while maintaining the temperature at 0°C–5°C with continuous stirring; to the reaction mixture, benzoyl chloride was added dropwise and the reaction was continued **(** Figure 27 **)**. The characterization of the compounds was done by spectral analysis, and an *in-vivo* study was done using the MES and PTZ methods. Three compounds showed significant anticonvulsant activity while one named 4-[5-benzoyl amino-(1,3,4)-thiadiazole-2yl-sulfanyl]-benzene sulfonyl chloride out of the three showed the best activity at 25 mg/kg (Pattanayak et al., 2009).

**FIGURE 28:**
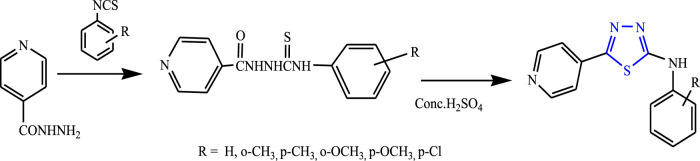
Synthetic pathway for synthesizing new phenyl isothiocyanate 1,3,4-thiadiazole derivatives.

Yar et al. reported a series of five-membered heterocyclic compounds with their anticonvulsant activity by refluxing isoniazid with equimolar substituted phenyl isothiocyanates using ethanol as a solvent for 5–6 h to form substituted phenylthiosemicarbazides, then H_2_SO_4_ was added, and continuous stirring was done at 0°C–5°C to form the main compound **(** Figure 28 **)**. The spectral analysis was done to confirm the structure of the compounds. 2-(4-Chlorophenyl) amino-5-(4-pyridyl)-1,3,4-thiadiazole was found to be highly potent during the evaluation by MES and PTZ methods as it contains the Cl group at the para position and showed 100% protection at a low dose (25 mg/kg) (Yar et al., 2009).

**FIGURE 29:**
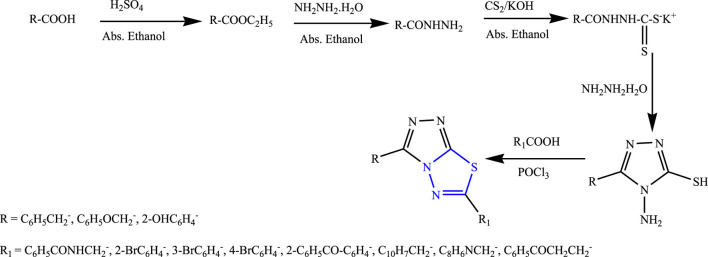
Systematic scheme for synthesizing targeted aryl-substituted 1,3,4-thiadiazole derivatives.

Twenty new derivatives of 1,3,4-thiadiazole were synthesized by Husain et al. The first esterification was done to form acid hydrazide, and then in the presence of potassium hydroxide, carbon disulfide, ethanol, and potassium dithiocarbamate were formed; further, 5 h reflux was done in the presence of aromatic acid to synthesize the main compounds **(** Figure 29 **)**. Spectral analysis was done to confirm the structure of the compounds, and their *in-vivo* anticonvulsant activity was checked by PTZ and MES models. Neurotoxicity was also determined taking phenytoin and carbamazepine as standard, out of which six compounds were found to be potent in the MES test while four compounds successfully passed the neurotoxicity test as the potent compounds contain halo-substituted aryl (bromophenyl) in the sixth position of the triazolothiadiazole ring which is essential for the anticonvulsant activity, as it showed great results at a low dose (30 mg/kg within ½ h) with no toxicity (Hussain et al., 2009).

**FIGURE 30:**
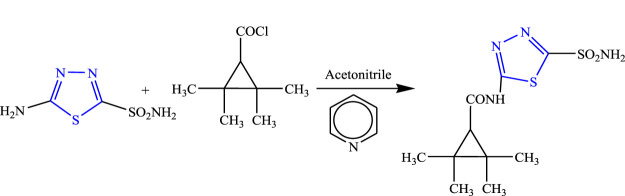
Synthetic pathway for synthesizing new 1,3,4-thiadiazole-2-sulfonamide derivatives.

5-(Tetramethyl cyclopropane carbonyl amido)-1,3,4-thiadiazole-2-sulfonamide was synthesized by Bialer et al. using pyridine as solvent **(** Figure 30 **)**. Spectral analysis was done to confirm the structure of the compound, and *in-vivo* anticonvulsant activity was checked by the PTZ and MES models, while neurotoxicity was checked using the rotarod method. The synthesized compound showed the highest activity only in the MES model with no toxicity, and ED_50_ was found at the lowest dose (16 mg/kg) within 30 min (Bialer et al., 2007).

**FIGURE 31:**
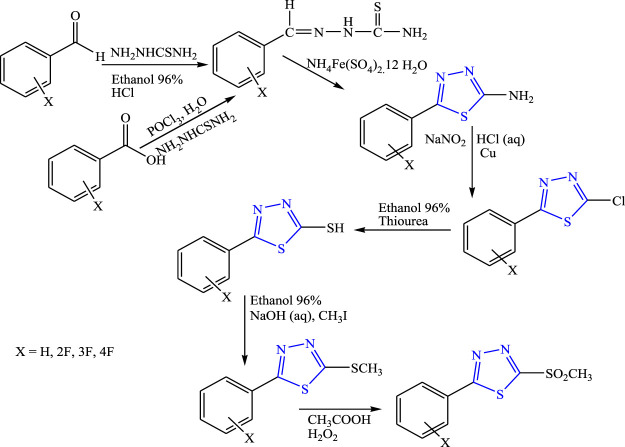
Synthetic pathway for synthesizing new 5-aryl-1,3,4-thiadiazole derivatives.

Foroumadi et al. synthesized 5-aryl-1,3,4-thiadiazole derivatives **(** Figure 31 **)**. Spectral analysis was done to confirm the structure of the compounds, and *in-vivo* anticonvulsant activity was checked by the PTZ method taking diazepam and flumazenil as standard. It is concluded that the amino-substituted compound (LD_50_ > 500 mg/kg) showed anticonvulsant activity while the ring substituted with other derivatives such as mercapto and methyl sulfone either showed no activity or occurrence of convulsion (Foroumadi et al., 2000).

### Structure–Activity Relationship

Literature survey revealed that the SAR of thiadiazole for anticonvulsant activity is due to the presence of = N–C–S– moiety and the strong aromaticity of the ring. Other than that, substitution with halo (Cl, Br, F), nitro, methyl group aldehyde, hydroxy, and unsubstituted compounds (compounds containing hydrogen) in the ring increases the anticonvulsant activity. An important role is played by lipophilic substitution and electron-withdrawing group on the anticonvulsant activity of the compound.

## Conclusion

Thiadiazole moiety is a five-membered ring containing sulfur and nitrogen atoms interconnected with two carbon atoms (electron-deficient), along with a lone pair of electrons, and have high thermotic stability and an electron deficiency. Several pharmacological activities are exhibited by the 1,3,4-thiadiazole derivative, and it is found to be potent against the anticonvulsant activity of *in vivo* animal models and MES, PTZ, and neurotoxicity models. The mechanism of action which is responsible for 1,3,4-thiadiazole to act as anticonvulsant agents is by preventing neurons firing in the brain by releasing the chloride ions due to the GABAA pathway. The compound which is substituted with an electron-withdrawing group showed good potency as compared to the electron-withdrawing group. 1,3,4-Thiadiazole can be a potent and effective moiety for anticonvulsant research.

## Abbreviation

Br, Bromine; Cl, chlorine; ED_50_, effective dose; F, fluorine; HPTLC, high-performance thin-layer chromatography; IR, infrared spectroscopy; LD_50_, lethal dose; MES, maximal electroshock method; nm, nanometer; NMR, nuclear magnetic resonance; OCH_3_, methoxy; PTZ, pentylenetrazole-induced seizure method; SAR, structure–activity relationship; TLC, thin-layer chromatography.
